# Extracranial Germ Cell Tumors in Children: A Comprehensive Study on Epidemiology and Treatment Outcomes in 543 Patients

**DOI:** 10.1007/s13193-025-02348-y

**Published:** 2025-06-02

**Authors:** Sajid S. Qureshi, Saiesh V Reddy, Maya Prasad, Badira C Parambil, Girish Chinnaswamy, Ramanathan Subramaniam, Venkata RM Gollamudi, Poonam K Panjwani, Mukta Ramadwar, Vasundhara Smriti, Akshay Baheti, Nehal Khanna, Siddhartha Laskar, Jiffmi Jose, Sanjay Talole

**Affiliations:** 1 Division of Paediatric Surgical Oncology, Department of Surgical Oncology, Tata Memorial Hospital and Advanced Centre for Training Research and Education in Cancer (ACTREC), Tata Memorial Centre, Ernest Borges Road, Parel, 400012 Mumbai, India; 2 Homi Bhabha National Institute (HBNI), Mumbai, India; 3 Division of Paediatric Oncology, Department of Medical Oncology, Tata Memorial Hospital and Advanced Centre for Training Research and Education in Cancer (ACTREC), Tata Memorial Centre, Mumbai, India; 4 Department of Pathology, Tata Memorial Hospital and Advanced Centre for Training Research and Education in Cancer (ACTREC), Tata Memorial Centre, Mumbai, India; 5 Department of Radiodiagnosis, Tata Memorial Hospital and Advanced Centre for Training Research and Education in Cancer (ACTREC), Tata Memorial Centre, Mumbai, India; 6 Department of Radiation Oncology, Tata Memorial Hospital and Advanced Centre for Training Research and Education in Cancer (ACTREC), Tata Memorial Centre, Mumbai, India; 7 Department of Biostatistics, Tata Memorial Hospital and Advanced Centre for Training Research and Education in Cancer (ACTREC), Tata Memorial Centre, Mumbai, India

**Keywords:** Germ cell tumors, Extracranial, Children, Epidemiology, Treatment

## Abstract

The objectives of this study are to investigate the demographic distribution of pediatric extracranial germ cell tumors and to evaluate treatment patterns and outcomes. We analyzed the medical records of 543 consecutive patients aged 0–15 years who were diagnosed with an extracranial germ cell tumor between January 2006 and December 2019. The peak age of presentation was at 1 year for both genders with a second peak at 14 years specifically for girls. An overall female predominance was noted, although there were variations in gender across different age groups. The 5-year overall survival rate for patients receiving complete curative treatment was 83%. Patients with gonadal tumors had a significantly greater probability of overall survival (87.6%) than did those with extragonadal tumors (75.6%) (*p* < 0.0001). Additionally, patients with head and neck tumors (53.3%), mediastinal tumors (52.3%), and choriocarcinoma histology (33.3%) had a lower likelihood of survival. Pediatric extracranial germ cell tumors predominantly affect children under 5 years of age, with a notable female predominance. These tumors demonstrate overall favorable outcomes with appropriate treatment.

## Introduction

Germ cell tumors (GCTs) are uncommon in children under the age of 15, accounting for approximately 3% of solid tumors in this age group [1]. These tumors are believed to stem from events occurring in utero and arise from primordial germ cells. They can manifest in various anatomical locations and exhibit a wide range of biological behaviors, from benign to malignant. With advancements in chemotherapy and surgical care, outcomes have improved significantly [1–3]. Epidemiological studies improve our understanding of the potential causes and development of diseases, as well as the diagnostic and therapeutic challenges they present. This knowledge can, in turn, inform public health efforts aimed at optimizing patient outcomes [4]. However, our country lacks comprehensive national statistics on GCTs due to inadequate reporting from cancer registries. Additionally, small sample sizes in previous studies limit effective data compilation and the establishment of clinical guidelines [5].

As the largest referral center for cancer in India, our institute receives patients from various regions of the country. This referral pattern may reflect the distribution of GCTs in India, if not the actual burden of the disease. Therefore, the aim of the present study was to evaluate the demographic, histopathological, and treatment profiles of consecutive children aged 0–15 years with extracranial GCTs registered over a 14-year period (2006–2019) at our institute.

## Material and Methods

**Fig. 1:**
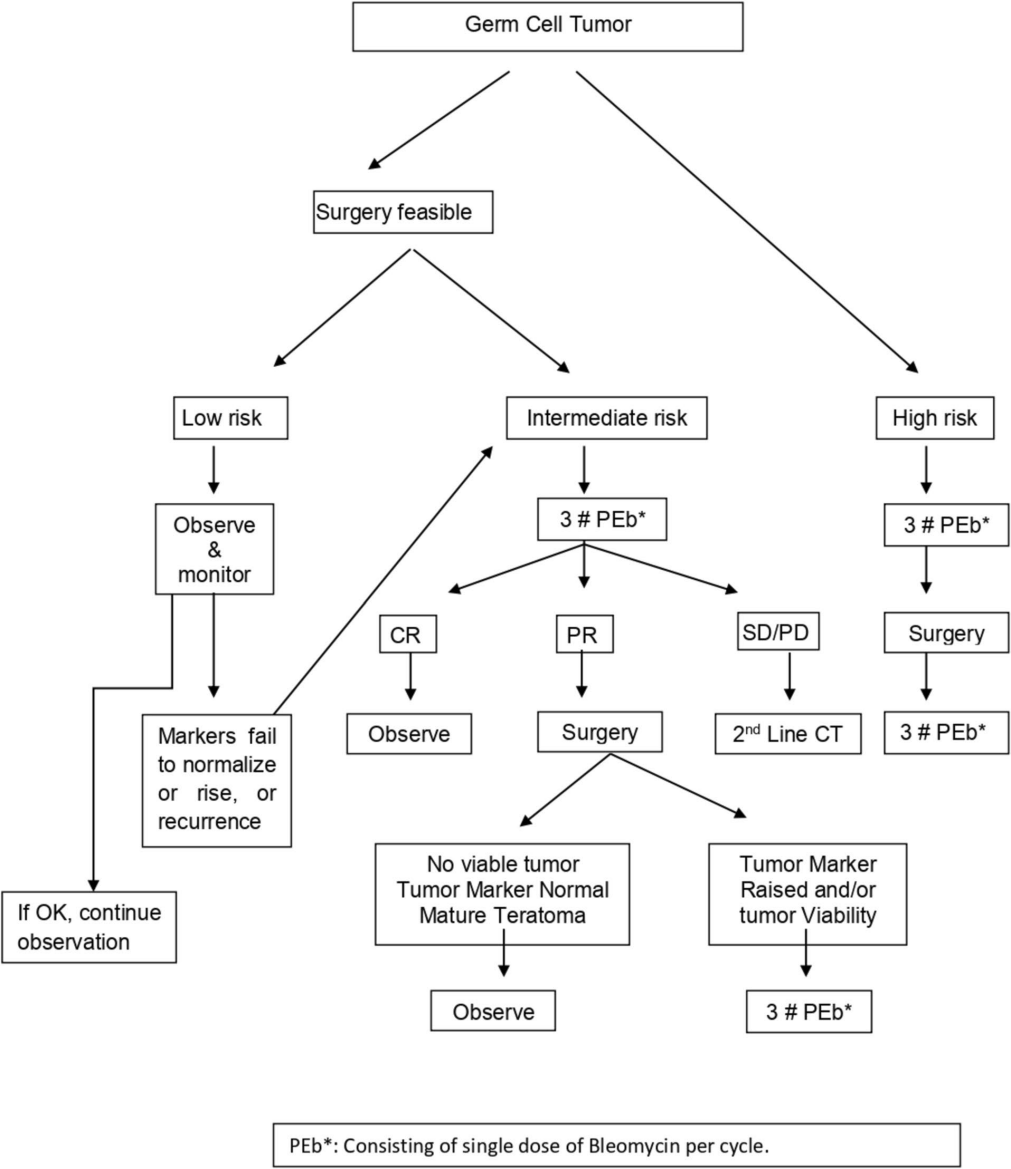
Management algorithm for pediatric extracranial GCTs

Approval from the Institutional Review Board and a waiver of consent were obtained for this retrospective study. We retrospectively evaluated all patients under the age of 15 who presented at Tata Memorial Hospital (TMH) with an extracranial benign or malignant GCT from January 2006 to December 2019. Both primary and recurrent cases were included in the analysis. Recurrent tumors were defined as new disease occurring after the completion of primary treatment or patients returning for care more than 6 months after their previous treatment. The diagnostic pathway for all patients included a comprehensive clinical evaluation and imaging studies, specifically a contrast-enhanced computed tomography (CECT) scan of the chest, abdomen, and pelvis, along with ultrasonography (USG) of the scrotum when indicated. Magnetic resonance imaging (MRI) was employed as necessary. Histological confirmation was obtained through assessment of resected samples following upfront surgery, with core biopsy or fine-needle aspiration cytology performed as appropriate. For patients who underwent biopsy or surgery at other facilities before presenting to the TMH, their histological samples were reviewed. Tumor markers, including serum alpha-fetoprotein (AFP) adjusted for age, beta-human chorionic gonadotropin (b-HCG), and lactate dehydrogenase (LDH), were evaluated in all patients at the time of presentation. Diagnosis of GCT was based on characteristic clinicoradiological features, histopathology, or elevated tumor markers. Patients who did not meet these criteria were excluded from the analysis. All treatment decisions were made during a tumor board meeting that included a team of specialists: pediatric medical and surgical oncologists, a radiation oncologist, a pediatric radiologist, a pathologist, a nuclear medicine specialist, and a palliative care physician. The therapeutic algorithm for treating malignant GCTs is illustrated in Fig. 1. The decision to address retroperitoneal lymph nodes and distant metastatic sites was individualized based on disease status and each patient’s condition. The treatment of both mature and immature teratomas primarily involves surgery, with coccygectomy being an integral part of the excision for sacrococcygeal GCTs. The preferred first-line chemotherapy regimen, known as PEb, consisted of cisplatin (33.3 mg/m^2^ per dose on days 1, 2, and 3), etoposide (167 mg/m^2^ per dose on days 1, 2, and 3), and bleomycin (15 units/m^2^ on day 1 only). The number of cycles administered (4–6) was determined based on risk stratification, dose density, response to treatment, and the presence of residual disease. Dose modifications and potential substitution of cisplatin with carboplatin were tailored to the patient’s fitness and tolerance. Subsequent salvage chemotherapy regimens for relapsed or refractory disease, including vinblastine, paclitaxel, and ifosfamide (TIP, TIC, VeIP, etc.), were personalized. Radiotherapy (RT) was employed in select patients with unresectable disease or patients with relapsed or refractory conditions.

### Treatment Groups

Treatment was categorized into four groups in accordance with the Consolidated Report of Hospital-Based Cancer Registries (2007–2011) published by the Indian Council of Medical Research [6].

- Prior treatment only (group 1): Patients who received some or complete cancer-directed treatment before registration and did not receive any further treatment at the TMH.
- Prior treatment and treatment at the TMH (group 2): Patients who received cancer-directed treatment before registration and then received further treatment at the TMH.
- Treatment only at the TMH (group 3): Patients presenting with or without a confirmed diagnosis of malignancy who had not received any cancer-directed treatment previously and received comprehensive cancer-directed treatment at the TMH.
- No cancer‐directed treatment (Group 4): This group included patients who neither received nor accepted any therapy. It also encompasses those who did not complete any form of treatment, where treatment status is unknown, and those who received only supportive care.

Following treatment, patients were followed up with clinical evaluations and tumor marker assessments every 3 months for the first 2 years, every 6 months during the next 3 years, and annually thereafter. Based on clinical suspicion, imaging studies such as CECT or USG of the abdomen and pelvis, along with chest radiographs, were conducted as needed. Patients who remained disease-free for more than 2 years from the completion of treatment were also encouraged to enroll in the after completion of treatment clinic at the institute for monitoring of growth and adverse effect of treatment.

### Statistical Analysis

Descriptive analysis was performed to evaluate age, gender, and the relative distributions of various histopathological subtypes using frequency distributions and percentages. Overall survival (OS) was calculated from the date of diagnosis to either death or the last follow-up. Survival analysis was conducted using the Kaplan–Meier method, with comparisons between groups made using the log-rank test. Statistical analysis was performed via IBM SPSS® Statistics 25.0 and R (version 4.0.5).

## Results

A total of 543 consecutive patients were eligible for the study. Among these, 497 patients presented with primary disease (91.5%), while 46 had recurrent disease (8.5%). During the study period, the average annual registration number of patients with extracranial GCTs was 39. The number of registrations increased more than fourfold, increasing from 15 patients in 2006 to 68 new registrations in 2019. The time trends during the study period are depicted in Table 1.

**Table 1:** Trends over time during the study period

	2006–2009	2010–2014	2015–2019
Registrations (*n*)	99	185	259
Gender Male Female	30 69	74 111	100 159
Age group > 5 5–10 > 10	45 11 43	113 18 54	128 34 97
Site Gonadal Extragonadal	67 32	96 89	158 101

### Age

**Fig. 2:**
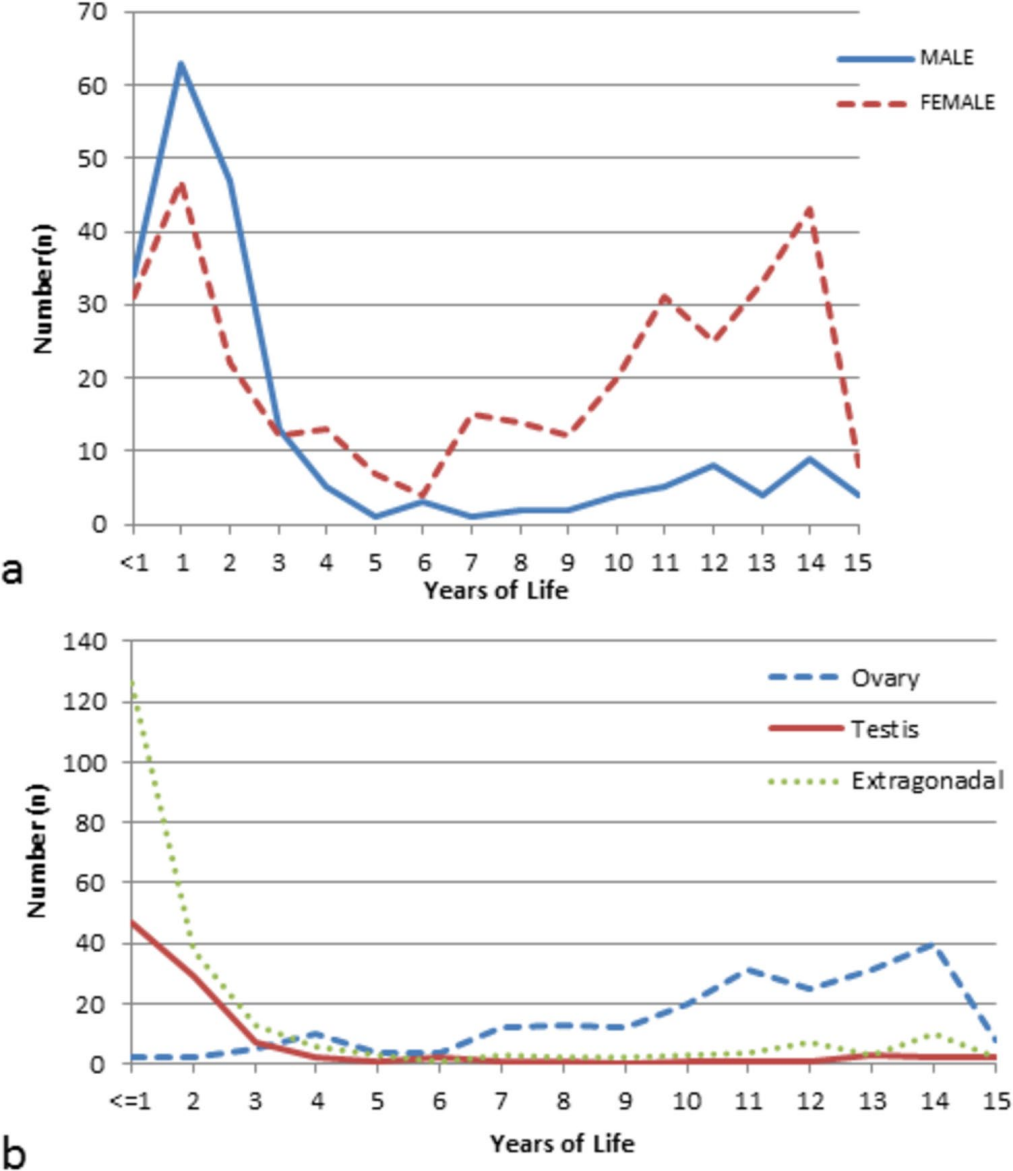
Gender and site distribution of germ cell tumors linked to age

The peak age for initial presentation is 1 year for both genders, with a notable second peak at 14 years for girls (Fig. 2). The median age at presentation was 4.1 years, with a range from 3 days to 15 years (Table 2). The most significant age disparity between genders was found in the gonadal group. The lowest median age, which was less than 2 years, was noted for extragonadal tumors, except for mediastinal tumors in boys.

**Table 2:** Age and gender distributions according to disease localization

	All sites (*n* = 543)			Gonads (*n* = 322)		Sacrococcygeal (*n* = 93)		Retroperitoneal (*n* = 50)		Mediastinal (*n* = 34)		Others (*n* = 44)	
	All *n* (%)	Boys *n* (%)	Girls *n* (%)	Boys *n* (%)	Girls *n* (%)	Boys *n* (%)	Girls *n* (%)	Boys *n* (%)	Girls *n* (%)	Boys *n* (%)	Girls *n* (%)	Boys *n* (%)	Girls *n* (%)
Median age (years) range	4.1 3 days–15 years	2 0.1–15 years	9.2 3 days–15 years	2 3–15 years	11.9 1.2–15 years	1.11 1–1.9 years	1.6 3 days–15 years	1.3 1–9.6 years	0.8 1–9.3 years	12.3 1.11–15 years	1.11 6–15 years	1.5 1–4.8 years	2 12 days–15 years
Age group < 5 years 5–10 years > 10 years	286 (52.6%) 63 (11.6%) 194 (35.7%)	161 (79%) 9 (4.4%) 34 (16.6%)	125 (36.8%) 54 (16%) 160 (47.1%)	85 (85%) 5 (5%) 10 (10%)	20 (9%) 47 (21.1%) 155 (70%)	24 (100%) 0 0	67 (97%) 1 (1.4%) 1 (1.4%)	28 (93.3%) 2 (6.6%) 0	17 (85%) 3 (15%) 0	3 (10.3%) 2 (6.8) 24 (82.7%)	3 (60%) 0 2 (40%)	21 (100%) 0 0	18 (78.2%) 3 (13%) 2 (8.6%)
Total	543	204	339	100	222	24	69	30	20	29	5	21	23

### Gender

Across all age groups, the majority of GCTs were diagnosed in girls, resulting in a male-to-female (M:F) ratio of 0.6:1 (Table 2). A male predominance was noted in children under 5 years, with an M:F ratio of 1:0.7. In contrast, a significant female predominance was found in children aged 5–10 years and those older than 10 years, with an M:F ratio of 0.2:1 (*P* < 0.0001). Among males, testicular tumors and extragonadal tumors were almost equally represented before age 5. However, extragonadal GCTs in females were predominantly diagnosed before age 5, while ovarian GCTs were the most common type diagnosed after age 10.

### Site

Gonadal tumors made up 59% of GCTs in children, primarily originating from the ovaries (see Tables 2 and 3). Rare sites for these tumors included the abdominal cavity, head and neck, and genitourinary region. Among children under 5 years of age, extragonadal tumors accounted for 63.2% (181 out of 286) of all GCTs. Among these, sacrococcygeal GCTs represented the largest subgroup, with 91 patients (32% of the total), and 74% of these patients were girls. For children aged 5 to 10 years and those older than 10, the ovaries were the most common site of disease, with 202 cases recorded. In children over 10 years old, mediastinal tumors were predominant, comprising 76.4% of cases, with a male-to-female ratio of 1:0.17.

**Table 3:** Histological characteristics and disease localization

	All sites (*n* = 543)			Gonads (*n* = 322)		Sacrococcygeal (*n* = 93)		Retroperitoneal (*n* = 50)		Mediastinal (*n* = 34)		Others (*n* = 44)	
	All *n* (%)	Boys *n* (%)	Girls *n* (%)	Boys *n* (%)	Girls *n* (%)	Boys *n* (%)	Girls *n* (%)	Boys *n* (%)	Girls *n* (%)	Boys *n* (%)	Girls *n* (%)	Boys *n* (%)	Girls *n* (%)
Yolk sac tumor	257 (47.3%)	135 (65.8%)	122 (36%)	79 (79%)	59 (26.5%)	15 (62.5%)	44 (63.7%)	13 (43.3%)	6 (30%)	14 (48.2%)	1 (20%)	14 (66.6%)	12 (52%)
Dysgerminoma/ seminoma	69 (12.7%)	3 (1.4%)	66 (19.4%)	0	65 (29.2%)	0	0	0	0	3 (10.3%)	0	0	1 (4.3%)
Mature teratoma	61 (11.2%)	26 (12.6%)	35 (10.3%)	6 (6%)	11 (5%)	5 (21%)	9 (13%)	9 (30%)	9 (45%)	3 (10.3%)	2 (40%)	3 (14.2%)	4 (17.3%)
Immature teratoma	49 (9%)	16 (7.8%)	33 (9.7%)	3 (3%)	19 (8.5%)	1 (4.1%)	5 (7.2%)	8 (26.6%)	5 (25%)	0	1 (20%)	4 (19%)	3 (13%)
Mixed histology	81 (15%)	19 (9.2%)	62 (18.2%)	9 (9%)	50 (22.5%)	3 (12.5%)	9 (13%)	0	0	7 (24%)	1 (20%)	0	2 (8.6%)
Choriocarcinoma	11 (2%)	2 (0.9%)	9 (2.6%)	1 (1%)	9 (4%)	0	0	0	0	1 (3.4%)		0	0
Embryonal carcinoma	8 (1.4%)	2 (0.9%)	6 (1.7%)	2 (2%)	4 (1.8%)	0	1 (1.4%)	0	0	0	0	0	1 (4.3%)
Others	7 (1.2%)	1 (0.4%)	6 (1.7%)	0	5 (2.2%)	0	1 (1.4%)	0	0	1 (3.4%)	0	0	0
Total	543	205	339	100	222	24	69	30	20	29	5	21	23

### Histology

Yolk sac tumors (YSTs) represented the most common histology, followed by teratomas (Table 4). Seminomas were the least common, with only three patients, all located in the mediastinum (Table 3). Dysgerminomas and YSTs were equally represented in ovarian cases. Teratomas were most frequently seen in children under 5 years of age and were the predominant histological subtype found in the retroperitoneal and head and neck regions. Choriocarcinoma was observed in children older than 5 years, with more than 80% arising in the ovary. Among children under 5 years of age, YST was the predominant histology (66%), whereas among those over 5 years of age, there was a relatively similar distribution of YST, dysgerminoma, and mixed malignant GCT.

**Table 4:** Histological characteristics according to age group

	< 5 years (*n* = 286)			5–10 years (*n* = 63)			> 10 years (*n* = 194)		
	All *n* (%)	Boys *n* (%)	Girls *n* (%)	All *n*(%)	Boys *n* (%)	Girls *n* (%)	All *n* (%)	Boys *n* (%)	Girls *n* (%)
Yolk sac tumor (*n* = 257)	189 (66%)	118 (72.2%)	71 (56.8%)	14 (22.2%)	4 (44.4%)	10 (18.5%)	54 (27.8%)	13 (38.2%)	41 (25.6%)
Dysgerminoma/ seminoma (*n* = 69)	2 (0.7%)	0	2 (1.6%)	17 (27%)	0	17 (31.4%)	50 (25.7%)	3 (8.8%)	47 (29.3%)
Mature teratoma (*n* = 61)	44 (15.3%)	22 (13.6%)	22 (17.6%)	5 (8%)	3 (33.3%)	2 (3.7%)	12 (6.1%)	1 (2.9%)	11 (6.8%)
Immature teratoma (*n* = 50)	29 (10%)	15 (9.3%)	14 (11.2%)	6 (9.5%)	1 (11.1%)	5 (9.2%)	14 (7.2%)	0	14 (8.7%)
Mixed histology (*n* = 81)	19 (6.6%)	6 (3.7%)	13 (10.4%)	18 (28.5%)	1 (11.1%)	17 (31.4%)	44 (22.6%)	12 (35.2%)	32 (20%)
Choriocarcinoma (*n* = 11)	0	0	0	3 (4.7%)	0	3 (5.5%)	8 (4.1%)	2 (5.8%)	6 (3.7%)
Embryonal carcinoma (*n* = 8)	2 (0.7%)	0	2 (1.6%)	0	0	0	6 (3%)	2 (5.8%)	4 (2.5%)
Others (*n* = 7)	1 (0.3%)	0	1 (0.8%)	0	0	0	6 (3%)	1 (2.9%)	5 (3.1%)
Total (*n* = 543)	286	161	125	63	9	54	194	34	160

### Treatment

Out of 497 patients with primary disease, 240 (48.2%) belonged to treatment group 2, while 223 (44.8%) belonged to group 3. Groups 1 and 4 included 19 (3.8%) and 15 (3%) patients, respectively. The majority of the earlier treatments in group 2 was surgery alone (*n* = 191, 80%), predominantly for testicular, ovarian, and sacrococcygeal tumors; revision surgery was needed in 24% (*n* = 46) of these patients. In group 3, patients underwent surgery either alone or in combination with chemotherapy (*n* = 193, 86.5%). Only six patients, all with mediastinal tumors that were not suitable for surgical resection, received RT alone as their local treatment. Chemotherapy-related mortality was observed in nine patients (4%). Patients presenting with recurrent tumors received salvage therapy, which included combinations of surgery, chemotherapy, and RT.

### Survival

**Fig. 3:**
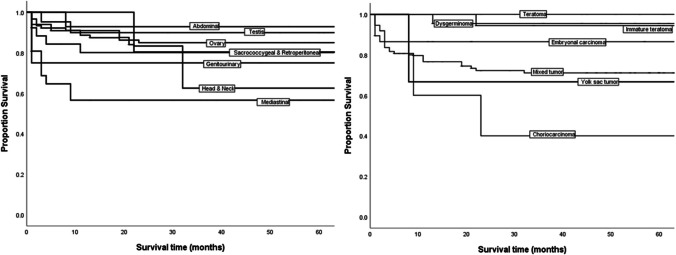
Overall survival linked to tumor subsites and histopathological types

At a median follow-up of 62 months (range 0.1 to19 years), the estimated 5-year OS for patients with primary disease receiving complete cancer-directed treatment at TMH was 83%. Patients with gonadal tumors had significantly better OS (87.6%) than those with extragonadal tumors (75.6%), with a *p*-value of < 0.004. Survival rates varied based on the tumor site: abdominal disease had an OS of 94.4%, retroperitoneal had 83.3%, and genitourinary GCTs had 85.7%. In contrast, lower OS rates were observed in sacrococcygeal (75.2%), mediastinal (52.3%), and head and neck (53.3%) GCTs, with a *p*-value of less than 0.0001 (Fig. 3).

Among different tumor histologies, mature teratomas (100%) and dysgerminomas (96%) had the best prognoses. Other histologies, including mixed tumors (82.6%), immature teratomas (91%), and embryonal carcinomas (85.7%), also demonstrated good OS rates. YST showed an OS of 77%, while the poorest outcome was seen in patients with choriocarcinoma, which had an OS of 33.3%, with a *p*-value of less than 0.0001.

## Discussion

Country-specific epidemiological data are essential for understanding the disease burden within a society. This information plays a crucial role in strengthening public health initiatives and enhancing referral centers to optimize treatment, reduce disability, and improve patient outcomes [4]. However, such reports are exceedingly scarce in our country, which has the second-largest population in the world. Previous studies have largely focused on gender-specific cases or malignant GCT [5]. The current study includes 543 patients under 15 years treated over 14 years and represents the largest single-center cohort of pediatric extracranial GCTs to date. In addition to presenting demographic profiles, tumor sites, and histopathological subtypes, this study also offers insights into the referral and treatment patterns of the institution. The most common age group at presentation in this study was children under 5 years. The median age at diagnosis varied depending on gender, tumor location, and type. In boys younger than 5 years, the most common tumor site was the testes, whereas in girls, the sacrococcygeal region was most common. In contrast, boys over 10 years of age predominantly had tumors in the mediastinum, and girls typically had ovarian tumors. The most common histological types in children younger than 5 years were YSTs and teratomas. In contrast, various histological types, including YST, mixed histology, and dysgerminomas, were observed in children older than 5 years. These differences support the hypothesis that GCTs in early childhood are biologically distinct from those occurring in older children [2]. In boys, germ cells undergo mitotic proliferation before and after birth, whereas in unborn girls, these cells undergo meiotic arrest and are reactivated only at puberty [2].

In this study, gonadal tumors accounted for 60% of the cases, outnumbering extragonadal tumors, which accounted for 40%. This may represent a relative increase, as intracranial GCTs were excluded from the analysis. The most common extragonadal site was the sacrococcygeal region, while the ovaries were the predominant site for gonadal tumors. A similar distribution has been reported in prior studies [2, 7, 8]. The histological type most commonly observed was YST, with teratomas being the second most common type of GCT in this study. This finding contrasts with earlier research, including studies from India, where teratomas constituted a significant portion of the cases [2, 7, 8]. Since teratomas are typically treated surgically at the primary institution, they are not usually referred for treatment, which may explain the discrepancy in our findings. Nonetheless, teratomas at complex sites, such as the retroperitoneum and head and neck region, were frequently observed in the present cohort of patients.

This study examines the referral and treatment patterns of GCTs at the author’s institution. A significant majority of the patients (92%) presented in the primary care setting. Among these patients, 52% had previously received treatment, primarily surgical interventions for gonadal and sacrococcygeal tumors, at other facilities before arriving at the TMH, which aligns with our previous findings [9]. However, nearly 25% of these patients require additional surgical procedures despite having undergone prior surgeries. The ease of accessing gonadal and sacrococcygeal sites, compared with more complex areas such as the retroperitoneum and mediastinum, which typically require expertise from high-volume centers, may explain why patients undergo initial surgeries before being referred. Nevertheless, a lack of surgical precision regarding the timing, approach, and extent of the initial procedures resulted in incomplete surgeries at the primary level, leading to the need for revision surgeries. Except for six patients with unresectable mediastinal GCTs, all patients who received complete treatment underwent surgery at the author’s institution. Treatment abandonment occurred in 7% of patients for various reasons, with nearly half of these individuals not receiving any form of cancer-directed treatment. This highlights the urgency for a coordinated effort in the medical and public health sectors to identify the factors influencing treatment defaults and to implement solutions that optimize patient outcomes. The estimated 5-year OS rate for patients treated at the TMH is 83%. While this rate includes all stages and sites of GCTs, it reflects the favorable outcomes and high cure rates documented in earlier studies [1–3]. Consistent with previous research, extragonadal GCTs demonstrated poorer outcomes, particularly in the mediastinal and head and neck regions [7]. Additionally, patients with more aggressive histological types, such as choriocarcinoma, have lower survival rates.

However, this study has several limitations. As a tertiary referral center, Neyman’s bias may affect the reported outcomes, particularly the relatively small number of teratoma cases. The age limitation of 15 years inadvertently excluded the adolescent age group between 15 and 19 years, who have a significant incidence of GCT. This also may affect the distribution and outcome patterns of the cohort. The deficiency in stage distribution and relapse patterns further hinders a comprehensive understanding of the disease. Furthermore, treatment-related toxicity and late effects of therapy may have provided additional insights into the management challenges faced with this rare group of tumors. Despite these limitations, this study contributes to the national statistics on GCTs and aims to encourage both national and international collaboration to refine and standardize management strategies for pediatric GCTs.

In conclusion, pediatric extracranial GCTs predominantly affect girls under 5 years of age, with gonadal sites being the most commonly involved. YST is the most prevalent histology. Surgery and chemotherapy remain the mainstays of treatment, achieving favorable survival rates at high-volume referral centers. There is a need for further prospective and collaborative studies to address gaps in epidemiological trends and management strategies, ultimately optimizing patient outcomes.

## Data Availability

Data available on request due to privacy/ethical restrictions.
